# Feature Detection in Visual Cortex during Different Functional States

**DOI:** 10.3389/fncom.2017.00021

**Published:** 2017-04-20

**Authors:** Pavel Esir, Alexander Simonov, Misha Tsodyks

**Affiliations:** ^1^Department of Neurotechnologies, Lobachevsky State University of Nizhny NovgorodNizhny Novgorod, Russia; ^2^Department of Theory of Oscillations and Automatic Control, Radiophysics Faculty, Lobachevsky State University of Nizhny NovgorodNizhny Novgorod, Russia; ^3^Department of Neurobiology, Weizmann Institute of ScienceRehovot, Israel

**Keywords:** synaptic plasticity, ring model, visual cortex, rate model, feature detection

## Abstract

Cortical activity exhibits distinct characteristics in different functional states. In awake behaving animals it shows less synchrony, while in rest or sleeping state cortical activity is most synchronous. Previous studies showed that switching between functional states can change the efficiency of flowing sensory information. Switching between functional states can be triggered by releasing neuromodulators which affect neurotransmitter release probability and depolarization of cortical neurons. In this work we focus on studying primary visual area V1, by using firing rate ring model with short-term synaptic depression (STD). We show that reconstruction of visual features from V1 activity depends on the functional state, with best precision achieved at the state with intermediate release probability. We suggest that this regime corresponds to the state of maximal visual attention.

## 1. Introduction

Numerous experimental observations show that during different functional states cortex generates different activity dynamics, characterized in particular by different degree of syncrhonization. To a large extent, cortical functional state depends on whether the animal is awake and engaged in solving a task or it is quiet or sleeping (Poulet and Petersen, 2008; Okun et al., 2010; Harris and Thiele, 2011).

Transition from one cortical state to another can be regulated by releasing neuromodulators such as acetylcholine (ACh), noradrenaline (NA) or others. These neuromodulators were shown to reduce neurotransmitter release probability by influencing presynaptic calcium channels while simultaneously reducing postsynaptic potassium conductance, enhancing depolarization (McCormick et al., 1993; Giocomo and Hasselmo, 2007). It was shown that in the case when animal is behaviorally engaged, cortical activity is asynchronous and vice versa in quiet state. Computational modeling demonstrated that in clustered networks, resembling cortical connectivity, information flow is most efficient in the regime of intermediate synchronization (Mark and Tsodyks, 2012).

In this work we focus on studying how information processing in primary visual area V1 depends on cortical state. The main motivation for choosing V1 for this study is the fact that it is widely believed to be responsible for processing simple features of visual stimuli, in particular the orientation of stimulus edges. Pioneering experiments in studying information processing in visual cortex performed by Torsten Wiesel and David Hubel on cats revealed that when a bar of light with specific angle is presented to the animal, neurons in V1 responsible for detecting this angle fire with highest rate (Hubel and Wiesel, 1959). Moreover, neurons from the same cortical column tend to prefer similar orientations, and a set of neighboring columns, called “hypercolumn,” encode all possible orientations.

To account for experimentally observed independence of width of neuronal orientation tuning curves from the contrast of the stimulus, firing rate ring model (further for simplicity called ring model) was proposed in Ben-Yishai et al. (1995), Hansel and Sompolinsky (1998). The ring model consists of a network of selectively interconnected units described by their firing rates, each of which representing an average activity of a single V1 column. Units are arranged on a ring and each unit has its preferred angle. Connections are chosen in such a way that units with similar preferred orientation (PO) excite each other stronger, while units with opposite PO inhibit each other. This kind of network architecture could also be applied to different spiking models as in Smirnova and Chizhov (2011), Lajoie and Young (2016).

In the current study, we consider effects of short-term synaptic depression (STD) on information processing in V1 (Tsodyks et al., 1998; Romani and Tsodyks, 2014). Following Mark and Tsodyks (2012), we emulate changing of functional states by varying neurotransmitter release probability and depolarization level of cortical neurons. Since release probability is a crucial determinant of synaptic depression (Tsodyks and Markram, 1997), changing release probability is a plausible mechanism by which transitions between different cortical states could be controlled. To characterize the efficiency of visual information processing, we simulate the network receiving oriented stimulus and compute the precision with which the orientation of the stimulus can be read out from the activity of the network. We show that precision is optimal at a certain intermediate value of release probability, i.e., there is an optimal cortical state. Moreover, the optimal state is different for different strengths of external inputs, such that for stronger input, smaller release probability is optimal. These results could potentially be tested experimentally.

## 2. Methods

### 2.1. Model of cortical hypercolumn

The dynamics of cortical hypercolumn in visual area was described by ring model (Ben-Yishai et al., 1995; Hansel and Sompolinsky, 1998), which is rate model (Wilson and Cowan, 1972; Equation 1) with special connectivity matrix described by the (Equation 2) below. We also introduce short term depression as in (Tsodyks et al., 1998; Romani and Tsodyks, 2014; Equation 3).

(1) $$\begin{array}{l} \tau \frac{m_{i}(t)}{dt}=-m_{i}(t)+g(\left[\sum_{j\;=\;1}^{N}W_{ij}Ux_{j}(t)m_{j}(t)\right]\\ \;+\;I_{i}^{inp}(t)+I_{i}^{noise}(t)+I_{0}) \end{array}$$

$$\begin{array}{r} g\left(y\right)=\log\left(1+\exp\left(y\right)\right) \end{array}$$

Here *m*_*i*_(*t*) is a mean firing rate of the unit *i*, τ is time constant, *g* is a gain function, *I*_0_ is a mean depolarization level. Each i-th unit has its preferred angle $\theta_{i}=i\frac{\pi }{N}$ which corresponds to preferred orientation (PO). *W*_*ij*_ is the weight between i-th and j-th units, given by Equation (2), it includes homogeneous inhibitory part *J*_0_ and spatially modulated part *J*_1_cos(2(θ_*i*_−θ_*j*_)) which represents stronger connectivity between units sharing similar POs. *N* is the number of units.

(2) $$\begin{array}{r} W_{ij}=J_{0}+J_{1}\cos\left(2\left(\theta_{i}-\theta_{j}\right)\right) \end{array}$$

*U* is a baseline level of neurotransmitter release probability. τ_*rec*_ is a time constant for neurotransmitter recovery. *x*_*i*_(*t*) is an amount of neurotransmitter in presynaptic unit *i*, governed by the following equation:

(3) $$\begin{array}{r} \frac{x_{i}\left(t\right)}{dt}=\frac{1-x_{i}\left(t\right)}{\tau_{rec}}-Ux_{i}\left(t\right)m_{i}\left(t\right) \end{array}$$

$I_{i}^{inp}\left(t\right)$ is the external stimuli input for unit *i*, with amplitude *C* and duration *T*. Angles $\theta_{k}^{inp}$ of external stimuli are uniformly distributed between 0 and π. rect is rectangular function, (Equation 5). *N*_*stim*_, *t*_*k*_ stand for the number and times of stimuli, respectively:

(4) $$\begin{array}{r} I_{i}^{inp}\left(t\right)=C\overset{N_{stim}}{\underset{k}{\sum }}\text{rect}\left(\frac{t-t_{k}}{T}\right)\cos\left(2\left(\theta_{k}^{inp}-\theta_{i}\right)\right) \end{array}$$

(5) $$\begin{array}{r} \text{rect}\left(\frac{t-t_{k}}{T}\right)=\left\{ \begin{array}{cc} 1\; & \text{if }t_{k}<t<t_{k}+T\\ 0\; & \text{elsewhere}\\ \; \end{array}\right. \end{array}$$

Finally, $I_{i}^{noise}\left(t\right)$ is a colored noise with standard deviation σ and correlation time τ_*n*_, each unit receives noise uncorrelated with others:

(6) $$\begin{array}{r} \tau_{n}\frac{dI_{i}^{noise}\left(t\right)}{dt}=-I_{i}^{noise}\left(t\right)+\sigma \sqrt{2\tau_{n}}\xi_{i}\left(t\right)\\ \langle \xi_{i}\left(t\right)\xi_{j}\left(t^{\prime }\right)\rangle =\delta \left(t-t^{\prime }\right)\delta_{ij} \end{array}$$

Parameters for simulations are shown in the Table 1. These parameters are used in all simulations unless otherwise stated.

**Table 1:** **Parameter values for simulations**.

**Parameter**	**Value**
*J*_0_	−12
*J*_1_	30
τ	0.01 s
τ_*rec*_	0.8 s
τ_*n*_	0.1 s
σ	2
*N*	200
*C*	5, 10, 20, 40
*T*	0.05 s, 0.2 s
*Freq*	4 Hz
*T*_*sim*_	2,000 s

Transitions between functional states were implemented by simultaneously changing *U* and *I*_0_ while keeping mean firing rate ⟨*m*⟩ ≈ 0.5 Hz, (see Equation 7). *T*_*sim*_ is total simulation time. Corresponding values of *I*_0_ for each *U* are shown in **Figure 2**.

(7) $$\begin{array}{r} \langle m\rangle =\frac{1}{T_{sim}N}\overset{T_{sim}}{\underset{0}{\int }}\overset{N}{\underset{i=1}{\sum }}m_{i}\left(t\right)dt \end{array}$$

### 2.2. Readout

In order to analyse the detection precision of orientations of external stimuli we calculate the so called Population Vector (PV), (Equation 8). The PV is a complex number which represents the angle of activity bump (argument of PV) and its magnitude (absolute value of PV). Readout was performed with variable numbers of neurons *N*_*read*_. Readout uses a sample of units than are involved in processing of stimuli, reflecting sparse connectivity between different regions of the cortex (Schüz, 2003).

(8) $$\begin{array}{r} \tau_{r}\frac{dR\left(t\right)}{dt}=-R\left(t\right)+\frac{1}{N_{read}}\overset{N_{read}}{\underset{j=1}{\sum }}\exp\left(-2\text{i}\theta_{j}\right)\chi_{j}\left(t\right), \end{array}$$

where *R*(*t*) population vector, χ_*j*_(*t*) is a number of spikes emitted by neuron *j*, chosen randomly with Poisson distribution those probability mass function is $P\left(k\right)=\frac{{\left(m_{j}\left(t\right)\right)}^{k}}{k!}e^{-m_{j}\left(t\right)}$.

To distinguish the errors caused by sparseness and discreteness of readout and the errors caused by moving of bumps we also calculate PV, according to Equation (9). ER (Exact Readout) can be considered as 2nd coefficient of spatial Fourier expansion of activity *m*_*j*_(*t*).

(9) $$\begin{array}{r} ER\left(t\right)=\frac{1}{N}\overset{N}{\underset{j=1}{\sum }}\exp\left(-2\text{i}\theta_{j}\right)m_{j}\left(t\right) \end{array}$$

For each stimulus deviation of detected angle (argument of *R*(*t*) and *ER*(*t*)) from the true angle of the stimulus was calculated and averaged to estimate detection error (Equation 10). In error estimation we also introduce a delay (*lag*), because it takes time for readout neurons to react. Hence, for each functional state controller by *U, I*_0_ and readout sparseness *N*_*read*_ we find *lag* for which detection error is minimal.

(10) $$\begin{array}{r} Errs\left(lag\right)=\frac{1}{TN_{stim}}\overset{N_{stim}}{\underset{k=1}{\sum }}\overset{t_{k}+T}{\underset{t_{k}}{\int }}\left|angle\left(R\left(t+lag\right)\right)-\theta_{k}^{inp}\right|dt \end{array}$$

### 2.3. Simulation tools

Dynamical system has been integrated by Euler-Maruyama method for SDE with 0.002 s time step. Calculation script was written in Python RRID:SCR_008394 with the help of numpy RRID:SCR_008633 and matplotlib RRID:SCR_008624; (Hunter, 2007) libraries. To run calculation for different parameters in parallel GNU parallel utility has been used (Tange, 2011). Scripts can be downloaded from github repository https://github.com/esirpavel/ring_plasticity_V1_fcn.

## 3. Results

### 3.1. Activity without external stimuli

In recurrent networks with ring architecture, spatially tuned activity can appear if self-excitation is strong enough (Ben-Yishai et al., 1995; Hansel and Sompolinsky, 1998). Further we will call such kind of activity “bumps.” When effects of synaptic plasticity are taken into account, repertoire of possible activities becomes richer and can lead to generation of waves, or spontaneous bumps of activity propagating short distances and then disappearing (York and van Rossum, 2009; Romani and Tsodyks, 2014).

Experimental evidence indicates that mean firing rate remains approximately constant for different behavioral states if no sensory stimuli is presented (Poulet and Petersen, 2008). To achieve this for each value of the release probability *U*, we set such value of baseline depolarization *I*_0_ that mean firing rate (Equation 7) remains approximately equal to 0.5 Hz in the absence of external stimuli (*C* = 0). Varying *I*_0_ and *U* simultaneously emulates influence of acetylcholine (ACh) or other neuromodulators that can reduce release probability and at the same time enhance depolarization of membrane potential by changing conductance of potassium channels (McCormick et al., 1993; Giocomo and Hasselmo, 2007).

**Figure 1:**
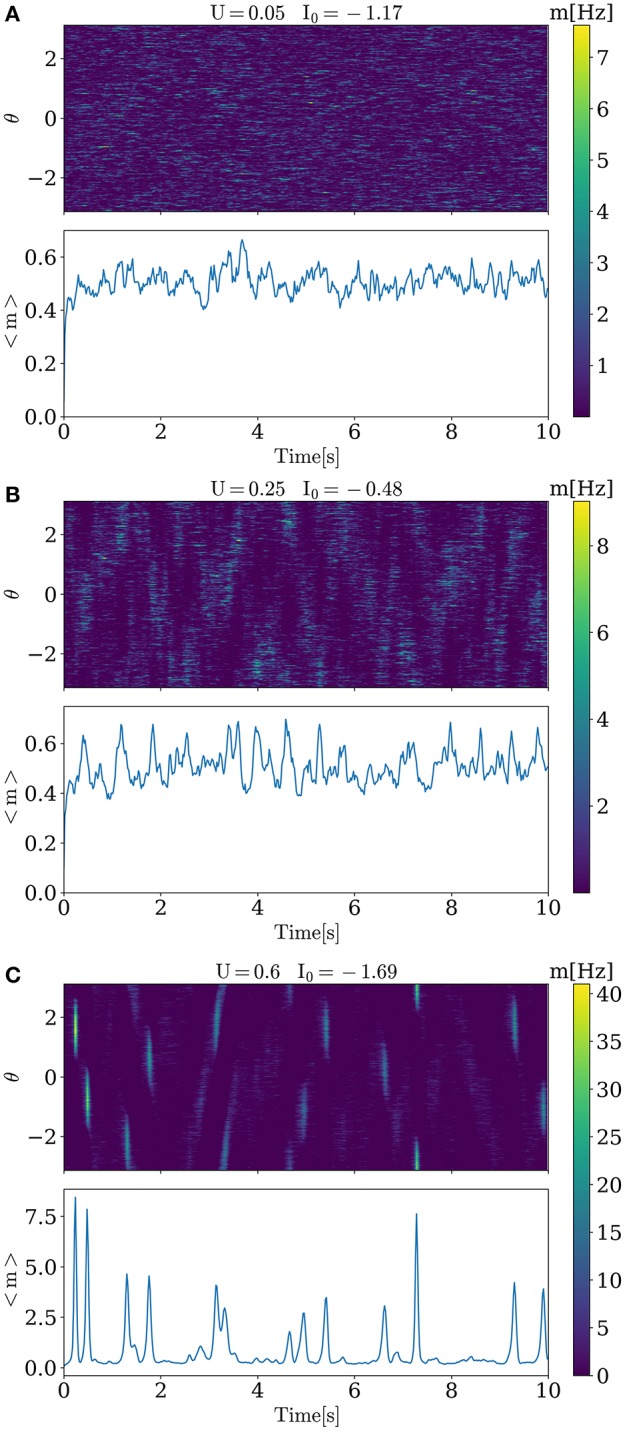
**Activity of the model for different values of ***U*** and ***I***_**0**_**. For low level of the release probability *U* = 0.05, small asynchronous fluctuations of activity are seen. For higher *U*, progressively stronger and more synchronous activations in the form of bumps emerge.

**Figure 2:**
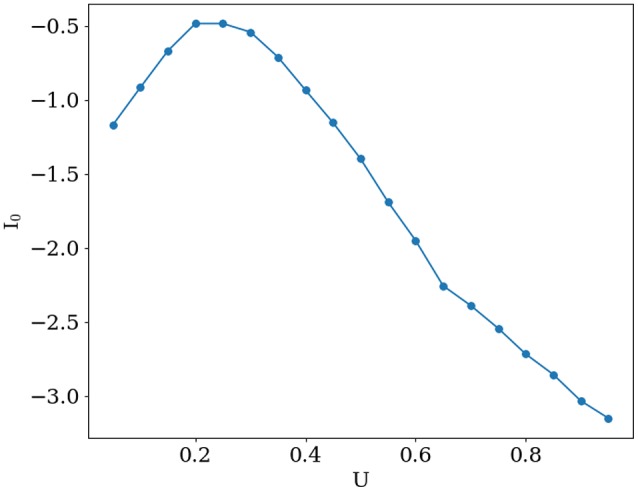
**Mean external input ***I***_**0**_ that keeps the mean network activity constant at ⟨***m***⟩ ≈ 0.5 Hz, as a function of ***U*****.

Activity for several characteristic values of *U* and *I*_0_ are shown in Figure 1. When increasing baseline release probability *U*, spontaneous bumps appear at *U* ≈ 0.4, further increasing their amplitude with growing *U*. All values of *I*_0_ and *U* that were used in simulations are shown in Figure 2. The positive slope in the beginning of the curve in this figure may appear paradoxical but can be understood as follows. When *U* is small, noise does not cause significant spatial fluctuations in the network activity, and the inhomogeneous input to the i-th neuron $r_{1}^{i}=J_{1}U\underset{j}{\sum }\cos\left(\theta_{i}\;-\;\theta_{j}\right)x_{j}\left(t\right)m_{j}\left(t\right)$ is smaller than homogeneous inhibition $r_{0}=J_{0}U\underset{j}{\sum }x_{j}\left(t\right)m_{j}\left(t\right)$, because convolution with cosine filters any spatially unmodulated input. Therefore, increasing U leads to decreasing of synaptic input to the neurons and hence for keeping the network activity at the same value we should increase *I*_0_. But, increasing *U* after reaching some intermediate values (in this case it is ≈ 0.2) noise starts to induce spatial fluctuations comparable with mean activity and hence excitation $r_{1}^{i}$ dominates over inhibition *r*_0_ because |*J*_1_| > |*J*_0_| (note than *J*_0_ is negative while *J*_1_ is positive). Thus, further increasing of *U* requires decreasing of the baseline input *I*_0_ to maintain the desired average network activity.

### 3.2. Activity with external stimuli

Each presented stimulus is characterized by time of occurrence and orientation angle. The input to each unit is set according to Equation (4), so units with preferred angles matching the orientation of the stimuli receive the strongest input with amplitude *C*. Duration of stimuli is *T*. Stimuli were presented as Poisson process with frequency *Freq* = 4 *Hz* and refractory period *T* in order to eliminate intersections. Thus, we do not model continuous presentation of stimuli, but only brief ones.

**Figure 3:**
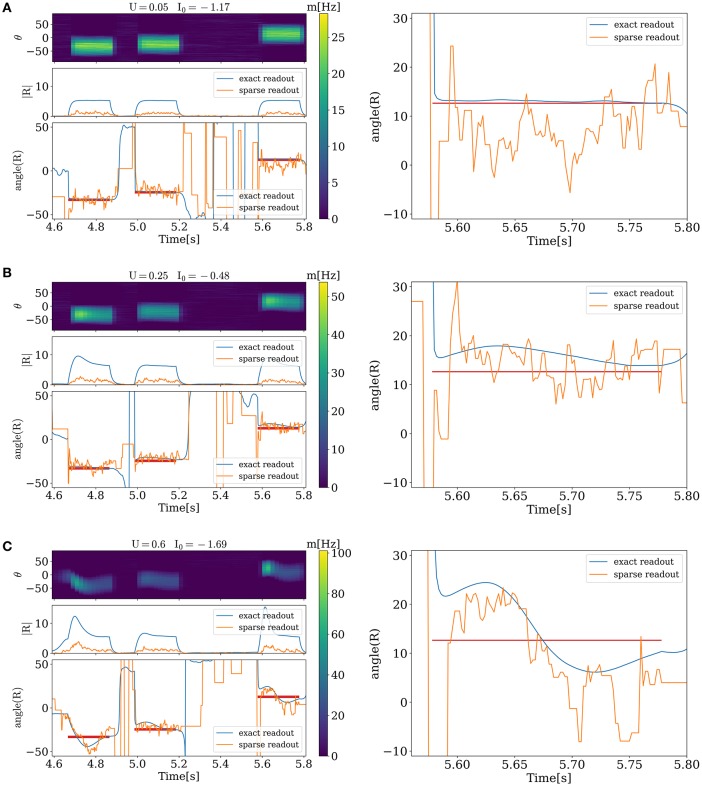
**Evoked activity of the network in the different functional states when external stimuli are presented**. On the left side, for each figure from top to bottom. Activity of each unit corresponding to its preferred angle, color code represents firing rate. Amplitude and angle (in degrees) of PVs, blue for exact readout and orange for sparse readout. On the right side zoomed dynamics of PV angle for exact and sparse readout. **(A)**, activity for minimal value of *U*. **(B)**, optimal state. **(C)**, activity when stimuli evoke waves. Duration, amplitude and frequency of stimuli *T* = 0.2 *s*, *C* = 20, *freq* = 4 *Hz*, *N*_*read*_ = 80.

Figure 3 illustrates the network activity in the presence of external stimuli. Each stimulus triggers a bump and changes the angle of PV toward the angle of the external stimulus (red horizontal lines on the left and right side). However, detected angles and applied ones do not match entirely. This happens because of two reasons. The first one is variability of neuronal response caused by discreteness and sparseness of readout; and the second one is intrinsic dynamics of the network. For small values of release probability firing rate is small and error caused by variability of neuronal response is large. It can be seen from Figure 3A, where sparse readout angle fluctuations around the stimulus are large, and at the same time exact population vector angle is very close to stimulus angle. For large values of *U* (Figure 3C) dynamics of the network becomes unstable and after presenting the stimuli a moving bump of activity can emerge, and it takes time before the angle detected by the network will settle down to the presented one. Between two of this regimes is an optimal state at which an error of sparse readout is minimal (Figure 3B).

### 3.3. Detection precision

**Figure 4:**
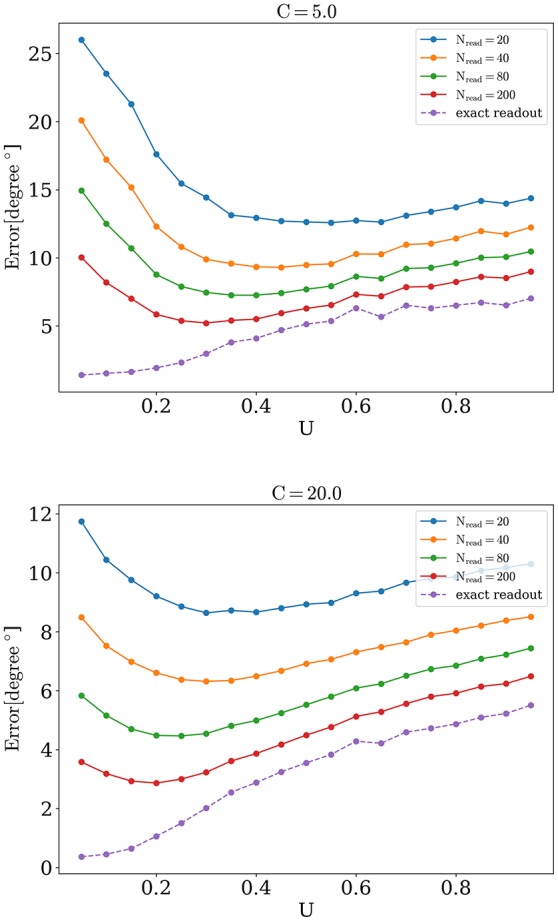
**Orientation detection error as a function of ***U*****.

**Figure 5:**
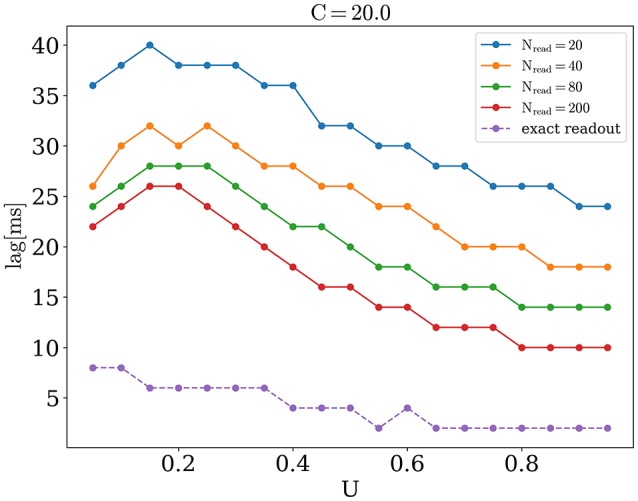
**Lags for optimal orientation detection as function of ***U***, for ***C*** = 20**.

We now address the main issue of the paper, namely the precision with which the network detects the orientations of external stimuli for different functional states. To this end, we calculated the error of detection according to Equation (10), for different values of the release probability *U* and numbers of readout neurons *N*_*read*_. Also we considered errors when PV was calculated according to Equation (9) (exact readout). The plots of the detection error vs. *U*, for small and large amplitudes of stimuli, are shown on Figure 4. On each graph, optimal detection for sparse readout is reached for some intermediate value of the release probability. Lags for minimal detection error for *C* = 20 are show on Figure 5. For bigger firing rates lags for optimal detection are smaller. Further all simulations was performed with *T* = 0.05 *s* and *freq* = 4 Hz. Simulations with longer and more frequent stimuli give qualitatively same results (data no shown).

**Figure 6:**
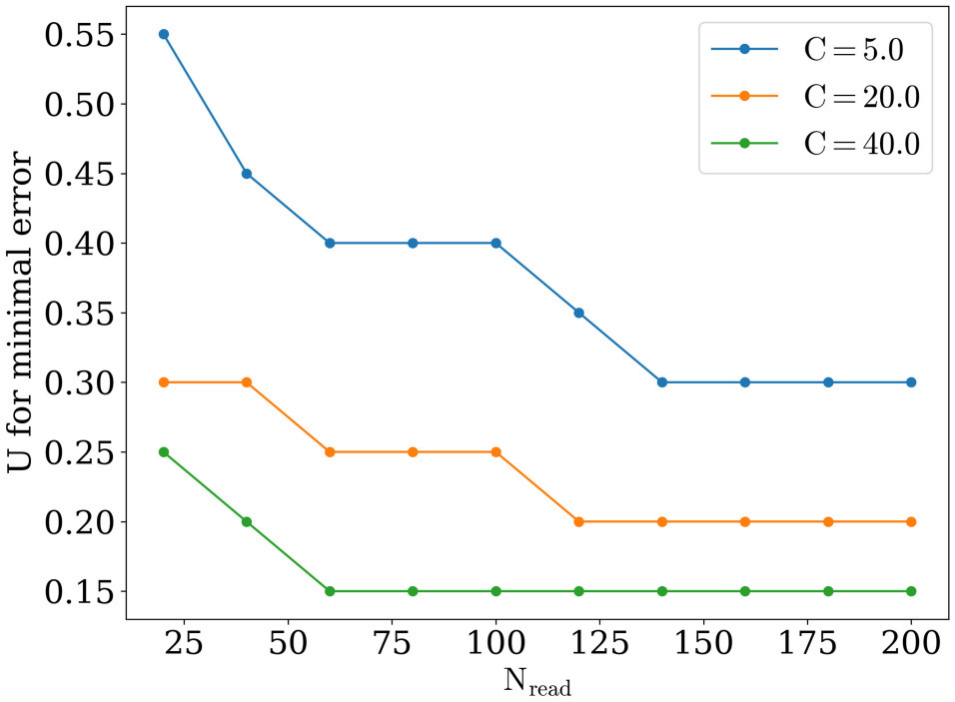
**Optimal value of ***U***, for different number of readout neurons and stimuli amplitude**.

The optimal value of *U* becomes lower while number of readout neurons is increased, similarly to what was reported in Mark and Tsodyks (2012). Also there is a tendency for the optimal *U* to be smaller for stimuli with higher amplitudes, as one can see in Figure 6. We discuss the implications of these observations below in Discussion section.

Appearance of optimal regime for orientation detection can be understood as follows. For large values of synaptic release probability, noise induces bigger fluctuations in network activity that in turn lead to moving bumps rotating away from the angle of the presented stimulus. This tendency is monotonic, as shown on Figure 4, dashed lines for exact readout. So firstly we have the tendency that smaller values of *U* are better for detection. But on the other hand, network with small values of release probability generates activity with lesser firing rate peaks and thus activity of readout neuron *R*(*t*) is more sensitive to noise, especially when *N*_*read*_ is relatively small. Competition between these two tendencies lead to the emergence of the optimal value of *U* for detection. According with the last tendency, the sparser readout is, the more dominating the effect of noise on readout, and hence the greater the optimal value of *U* for minimal error should be, consistently with our findings (Figure 6). Similar reasoning explains why increasing the amplitude of the stimuli should also result in smaller optimal values for release probability *U*, for bigger amplitudes, e.g., *C* = 40 and full readout, dependence of detection error from *U* becomes almost monotonically increasing as for exact readout (data not shown).

In Pinto et al. (2013) it was shown that optogeneric activation of basal cholinergic neurons or their axons in V1 improves visual discrimination in awake mice. Comparing with our finding, we can hypothesize that in normal conditions in quiet animal when sensory stimuli are sufficiently strong and baseline release probability is relatively big, for better performance when animal beginss to engage with environment, baseline release probability should be reduced by releasing of (ACh).

## 4. Discussion

We presented a model of a hypercolumn in the primary visual area V1, that is based on the well studied ring model (Ben-Yishai et al., 1995; Hansel and Sompolinsky, 1998). Here we focused in particular on the precision of orientation representation for different functional states of the cortical networks. To this end we considered the effects of synaptic depression in the intracortical connections on the precision of orientation representation, and assumed that cortical states are regulated via the effects of neuromodulators on synaptic release probability and depolarization of cortical neurons. We show that for different values of release probability and depolarization levels, network without external stimuli shows diverse spontaneous dynamics, beginning from asynchronous firing to generation of high synchronized activity in the form of bumps when release probability is high.

We estimated the precision with which network represents external stimuli. For different functional states regulated by release probability and depolarization level, network shows different precision. As reported in previous study, regime of intermediate synchrony is most preferable for optimal information flow (Mark and Tsodyks, 2012). Here we report that intermediate values of the release probability are also most preferable for orientation representation and discrimination in primary visual cortex. This follows from sparse connectivity between different regions of the cortex. We suggest that this regime correspond to the functional state of engagement with the visual stimulation. We also show that for different amplitude of external stimuli optimal value of *U* is different, namely the optimal value of *U* is smaller for stronger inputs. We hypothesize that cortical state in the brain are regulated in order to achieve the maximal performance according to external conditions.

An open question remains about the role of different types of inhibitory interneurons in the cortex, whose we did not took into account in our model, even though they play an important role in information processing in the cortex (Isaacson and Scanziani, 2011; Tremblay et al., 2016). For example in Lee et al. (2012) it was shown that activation of specific interneurons improves V1 feature selectivity and visual perception. Considering their influence to information processing in primary visual cortex will be the subject of future work.

## Author contributions

MT and PE designed the study; PE and AS performed all simulations; PE, AS and MT wrote the paper.

## Funding

This work excluding Section 3.3 was supported by The Russian Science Foundation No. 14-11-00693. A part of this work related to detection precision (Section 3.3) was supported by the grant (the agreement of August 27, 2013 No. 02.B.49.21.0003 between The Ministry of education and science of the Russian Federation and Lobachevsky State University of Nizhni Novgorod).

### Conflict of interest statement

The authors declare that the research was conducted in the absence of any commercial or financial relationships that could be construed as a potential conflict of interest.
